# Diagnostic value of MRS-quantified brain tissue lactate level in identifying children with mitochondrial disorders

**DOI:** 10.1007/s00330-016-4454-8

**Published:** 2016-06-07

**Authors:** Roelineke J. Lunsing, Kim Strating, Tom J. de Koning, Paul E. Sijens

**Affiliations:** 1 Department of Child Neurology, University Medical Centre Groningen, University of Groningen, Groningen, The Netherlands; 2 Department of Pediatric Metabolic Diseases, University Medical Centre Groningen, University of Groningen, Groningen, The Netherlands; 3 Department of Radiology, University Medical Centre Groningen, University of Groningen, Hanzeplein 1, 9713 GZ Groningen, The Netherlands

**Keywords:** Cerebrospinal fluid, Lactate, Magnetic resonance, MR spectroscopy, Mitochondri

## Abstract

**Objectives:**

Magnetic resonance spectroscopy (MRS) of children with or without neurometabolic disease is used for the first time for quantitative assessment of brain tissue lactate signals, to elaborate on previous suggestions of MRS-detected lactate as a marker of mitochondrial disease.

**Methods:**

Multivoxel MRS of a transverse plane of brain tissue cranial to the ventricles was performed in 88 children suspected of having neurometabolic disease, divided into ‘definite’ (n = 17, ≥1 major criteria), ‘probable’ (n = 10, ≥2 minor criteria), ‘possible’ (n = 17, 1 minor criterion) and ‘unlikely’ mitochondrial disease (n = 44, none of the criteria). Lactate levels, expressed in standardized arbitrary units or relative to creatine, were derived from summed signals from all voxels. Ten ‘unlikely’ children with a normal neurological exam served as the MRS reference subgroup. For 61 of 88 children, CSF lactate values were obtained.

**Results:**

MRS lactate level (>12 arbitrary units) and the lactate-to-creatine ratio (L/Cr >0.22) differed significantly between the definite and the unlikely group (p = 0.015 and p = 0.001, respectively). MRS L/Cr also differentiated between the probable and the MRS reference subgroup (p = 0.03). No significant group differences were found for CSF lactate.

**Conclusion:**

MRS-quantified brain tissue lactate levels can serve as diagnostic marker for identifying mitochondrial disease in children.

***Key points*:**

• *MRS*-*detected brain tissue lactate levels can be quantified*.

• *MRS lactate and lactate*/*Cr are increased in children with mitochondrial disease*.

• *CSF lactate is less suitable as marker of mitochondrial disease*.

## Introduction

Mitochondrial disorders represent a clinically, biochemically, and genetically heterogeneous group of diseases associated with dysfunction of the oxidative phosphorylation system (OXPHOS) [1]. Although most individual OXPHOS disorders are rare, epidemiological evidence suggests that the minimum birth prevalence is 1 in 7634 [2]. Curative treatment for the conditions remains elusive, and so does symptomatic treatment in most cases. Nevertheless, correct diagnosis of an OXPHOS disorder is important for prognosis, genetic counseling, and supportive management of associated impairments [3].

In mitochondrial disorders the process of adenosine triphosphate (ATP) production is disrupted. Low ATP results in an upregulation of glycolysis, leading to an overproduction of pyruvate, and this excess pyruvate is either transaminated to alanine or reduced to form lactate [4]. Venous lactate acidosis/lactic acidosis or elevated lactate is generally considered as a clinically relevant indicator for mitochondrial disease, but unfortunately can be falsely positive or negative. Cerebrospinal fluid lactate (CSF) may be elevated, even in the presence of normal venous lactate values [5, 6]. CSF lactate values may therefore be a more reliable diagnostic marker for a mitochondrial disorder than venous lactate in patients with neurological symptoms. Magnetic resonance spectroscopy (MRS) is a non-invasive functional brain imaging method that is capable of detecting biochemical metabolites in vivo. N-acetyl-aspartate (NAA) reduction and lactate accumulation in both the cerebral white and gray matter are the most prominent MRS signal abnormalities observed in mitochondrial disorders [7–14]. Lactate level elevation in the brain tissue of patients with mitochondrial disease, is even observed in the absence of systemic lactic acidosis [15].

The present study evaluated whether or not MRS brain tissue lactate values and CSF lactate values are good diagnostic markers for identifying children with mitochondrial diseases in a group of children suspected of having a neurometabolic disease. In our experience with multivoxel proton MRS [16], the summing of the MRS signals from multiple gray and white matter brain tissue voxels improves the detectability of metabolites owing to gains in the signal-to-noise ratio (SNR), which is proportional to total voxel volume provided that B_0_ and B_1_ homogeneity are such that line broadening is negligible [17–19]. A new aspect in this MRS study of brain lactate levels is that, compared with studies performed elsewhere, the signals in larger volumes of brain tissue are summed, allowing for better SNRs and thus quantitative assessment of lactate in children with or without neurometabolic disease.

## Materials and methods

### Subjects

This retrospective study was conducted at the University Medical Center Groningen, in accordance with the Declaration of Helsinki (Seoul, 2008). The requirement of informed consent was waived by the medical-ethical committee of the University Medical Center Groningen, since the study was retrospective and involved post-processing of clinical data.

From our medical records, we identified 96 patients under the age of 18 years in whom magnetic resonance imaging (MRI) including MRS was performed. These patients underwent MRI/MRS and a lumbar puncture, because of clinical suspicion of a neurometabolic disorder including mitochondrial disease, between January 2003 and July 2013. CSF lactate values and other information were obtained from patient files. Patients with disorders that may cause a lactate rise, like subarachnoid haemorrhage, meningitis, encephalitis, and ischemia, were excluded [20, 21]. Patients with seizures, frequent in mitochondrial disease (22), were included. Eight patients were excluded in the process of calculating the MRS data because of: (1) adding of the voxel signals was not possible (n = 2); (2) voxel measurements were not made in a standard area of the brain (n = 2) (data were not comparable, because different areas of the brain have different metabolite concentrations); (3) MRS data were lost (n = 2); (4) too much fluid in the volume of interest prohibited the selection of voxels mainly containing brain tissue (n = 1); and (5) poor spectra (n = 1) (poor resolution between metabolite peaks, probably because of movement of the patient during the MRS scan). In total, 88 patients were included. Median age at first MRS scan was 3.9 years (range 0 months to 15.2 years); there were four patients that underwent their first MRS scan at neonatal age (<2 months), 28 infantile (3 months to 2 years), 37 in early childhood (2–6 years) and 19 in late childhood (>6 years).

We constructed a scoring system to determine the likelihood of mitochondrial disease (Table 1). The mitochondrial disease scoring system was adapted from existing scoring systems [23] and based on several criteria including clinical features, MRI abnormalities associated with mitochondrial disease, muscle biopsy results (ATP production speed and enzymatic complex deficiencies), and genetic abnormalities associated with mitochondrial disease. We divided the subjects into four categories: 1 ‘Mitochondrial disease unlikely’, none of the criteria; 2 ‘Possible mitochondrial disease’, with one minor criterion i.e. ATP production rate <42.1 nmol/h.mUCS, compatible MRI abnormalities, or strong clinical suspicion; 3 ‘Probable mitochondrial disease’, ≥2 minor criteria, and 4 ‘Definite mitochondrial disease’, ≥1 major criteria, i.e. mitochondrial DNA mutation and/or enzymatic complex deficiency in muscle biopsy. From group 1 (‘mitochondrial disease unlikely’) we selected 10 children with a normal neurological exam in order to have the closest possible to a true control group (current guidelines do not allow for healthy infants serving as controls). This group is further referred to as the MRS reference subgroup. Using the mitochondrial disease scoring system 17 patients (19.3 %) scored a 4 ‘definite mitochondrial disease’, 10 patients (11.4 %) scored a 3 ‘probable mitochondrial disease’, 17 patients (19.3 %) scored a 2 ‘possible mitochondrial disease’, and 44 patients (50 %) scored a 1 ‘Mitochondrial disease unlikely’. Patient characteristics are listed in Table 2.

**Table 1:** Mitochondrial disease scoring

I. Minor diagnostic criteria
a) Abnormal muscle biopsy with lower mitochondrial energy production capacity
*ATP production speed* < *42.1 nmol*/*h.mUCS*
b) MRI abnormalities associated with mitochondrial disease
*Abnormality in the basal ganglia*, *the thalamus*, *mesencephalon and*/*or brainstem*, *and*/*or atrophy* (*for instance*, *abnormalities associated with mitochondrial encephalomyopathy*, *lactic acidosis*, *and stroke*-*like episodes* (*MELAS*) *or Leigh syndrome*)
c) Strong clinical suspicion of mitochondrial disease based on the experience of a team of child neurologists and metabolic pediatricians
*Symptoms associated with mitochondrial disease*: *therapy resistant status epilepticus*, *ophthalmoplegia*, *ptosis*, *retinopathy*, *hearing impairment*, *and movement disorders such as ataxia* (*without a known cause*), *pyramidal and*/*or extra pyramidal signs*, *and*/*or familial mitochondrial disease*
II. Major diagnostic criteria
d) DNA mutations associated with mitochondrial diseases
*Mitochondrial DNA mutations*, *core*-*bound DNA deletions* (*mitochondrial deletion syndrome*), POLG1* gene mutations and OPA1** *gene mutations*
e) Enzymatic complex deficiency
*Muscle biopsy*
1. Mitochondrial disease unlikely: None of the minor or major criteria are present.
2. Possible mitochondrial disease: One of the minor criterions is present.
3. Probable mitochondrial disease: Two or more minor criteria are present.
4. Definitive mitochondrial disease: One or more major criterion is present.

* POLG (alias, POLG1 or POLGa) is the gene that codes for the catalytic subunit of the mitochondrial DNA polymerase, called DNA polymerase gamma

**OPA1 is the gene that codes for the protein dynamin-like 120 kDa

**Table 2:** Patient characteristics

Category	N	Male/Female	Age (yr)	Venous lactate	CSF lactate	MRS lactate	MRS L/Cr
		(%)	(mean)	(mean^a^)	(mean^a^)	(mean; a.u.)	(mean)
Unlikely	44	68/32	4.6 (±4.1)	1.5 (±0.9)	1.5 (±0.3)	8.2 (±2.1)	0.17 (±0.04)
Possible	17	47/53	4.0 (±3.4)	2.1 (±1.5)	1.6 (±0.3)	8.9 (±3.6)	0.2 (±0.07)
Probable	10	60/40	2.3 (±1.6)	2.5 (±2.9)	2.3 (±2.4)	10.6 (±10.0)	0.23 (±0.08)
Definite	17	59/41	3.3 (±3.7)	3.7 (±3.0)	2.3 (±2.1)	11.2 (±5.5)	0.28 (±0.2)

N, number; yr, years; CSF, cerebral spinal fluid; MRS, magnetic resonance spectroscopy; L/Cr, lactate/creatine ratio; a.u., arbitrary units; ±, ± standard deviation of the mean; ^a^Values in mmol/l

### Methods

#### MRI/MR spectroscopy

**Fig. 1:**
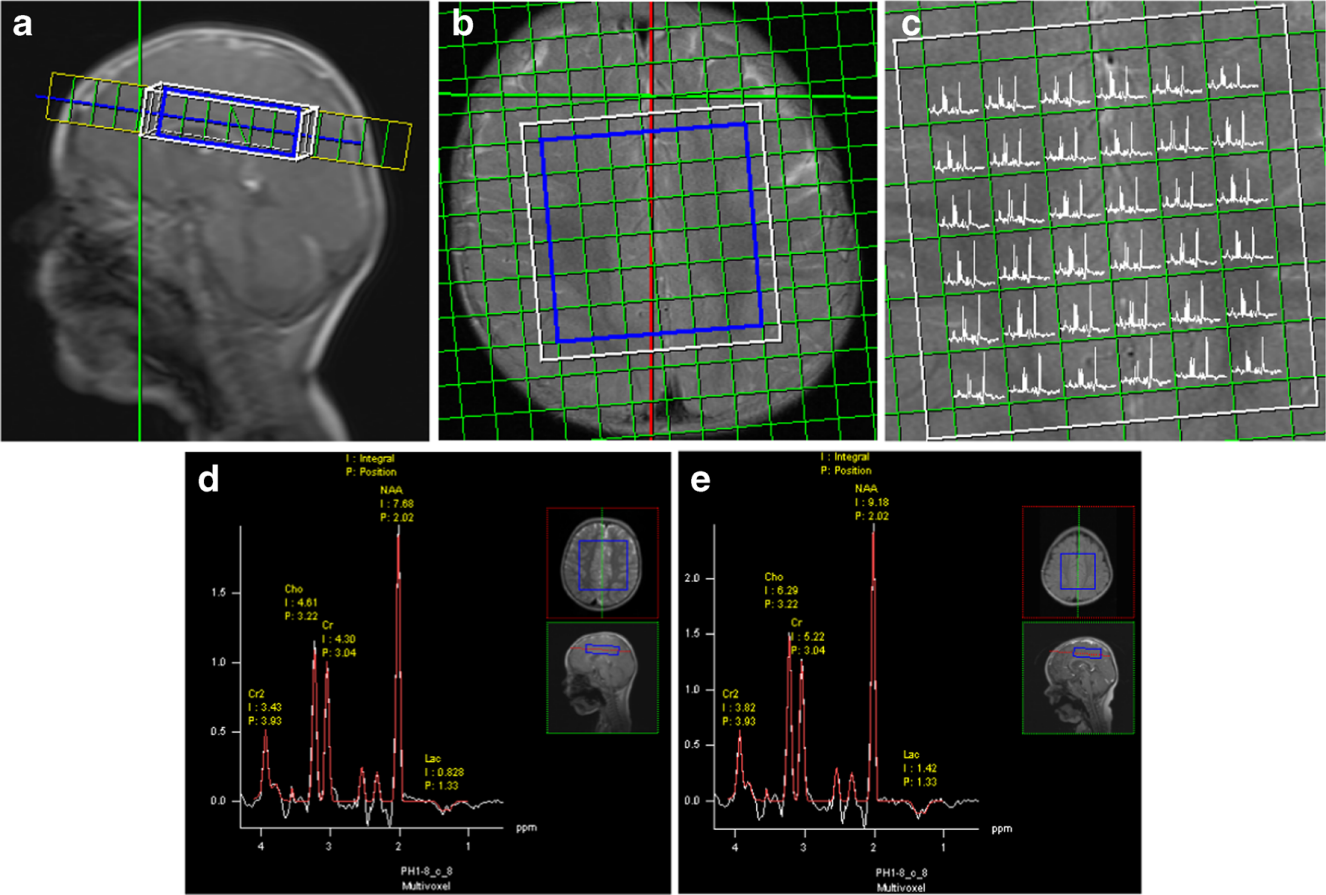
^1^HMR spectroscopy of a representative supraventricular region of interest (ROI), volume 7 × 7 × 2 cm^2^ (**a**, **b**). The resulting spectral map of the transverse plain, 36 voxels of 1 × 1 × 2 cm^2^ each (**c**). Representative summed spectrum for the entire ROI of a child from the unlikely group showing a modest lactate peak (the inverted doublet at 1.33 ppm) (**d**), and one of a child from the definite group showing increased lactate (**e**). p.p.m., parts per million

Pediatric brain MRI was performed using Siemens 1.5 T MR Scanners (subsequently the SP, Sonata, Avanto, and Aera product line), in a minority of cases under general anesthesia following institutional guidelines. The routine protocol included sagittal T1 weighted, axial T2-weighted, and axial fluid attenuated inversion recovery sequences that were used for multi-section spectroscopic imaging localization (Fig. 1). Two certified specialists, i.e. a pediatric neuroradiologist and a pediatric neurologist, that were blinded to the classification results interpreted the radiological studies in consensus. Imaging evaluation included: (1) ‘normal’; (2) ‘abnormality in the basal ganglia’; (3) ‘abnormality in the mesencephalon and/or brainstem’; (4) ‘atrophy’; (5) ‘abnormality in the thalamus’, and (6) ‘a combination of two or more of the possible abnormalities’ [2–5]. MRS studies were limited to our standard pediatric protocol of point-resolved spectroscopy (PRESS) combined with 2D chemical shift imaging (repetition time 1500 ms, echo time 135 ms) to obtain a transverse plane of typically 36 or 49 voxels of 1x1x2 cm^3^ each, located cranial to the ventricles [17]. Focal abnormalities, as may be present in different parts of the brain such as the basal ganglia, were therefore not featured. With this MRS method and using the manufacturer’s Syngo post-processing software, observer-independent automated metabolite peak areas are obtained for choline (Cho), creatine (Cr), N-acetylaspartate (NAA), inositol (Ins), and lactate (L) for an array of voxels containing gray and white matter [17]. In order to get adequate SNRs for the lactate peaks, MRS peak areas were summed for the entire region of interest of 36-49 voxels (54-73.5 cm^3^) and quantified. The results in arbitrary units, a.u., were standardized by referring to the unsuppressed water signal and also assessed relative to creatine (L/Cr).

### Reference values

**Fig. 2:**
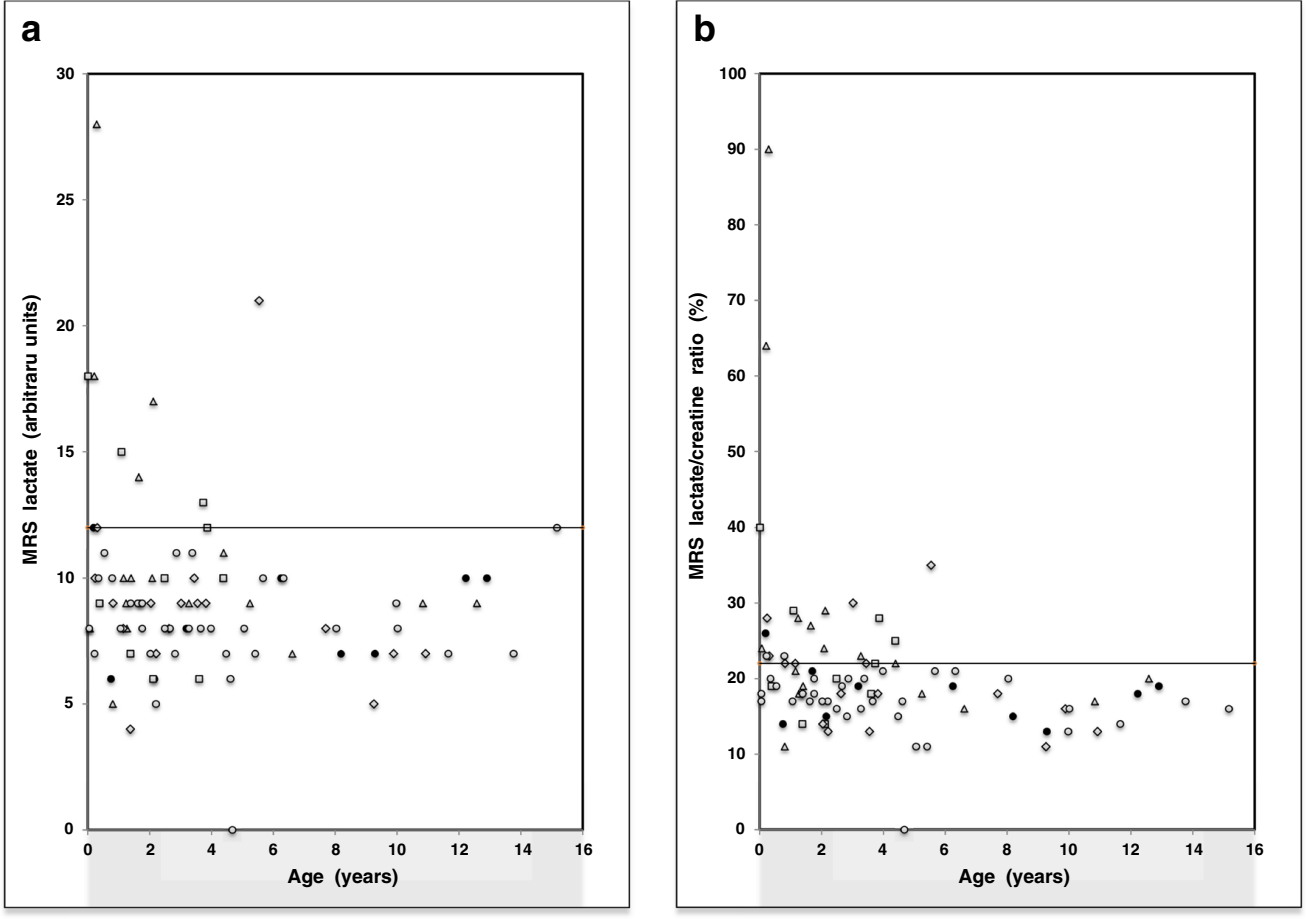
Scatter plot of the MRS lactate values (**a**) and percent L/Cr ratios (**b**) in the definite (Δ; n = 17), probable (□; n = 10), possible (◊; n = 17) and unlikely mitochondrial disease group (○,●; n = 44), and in a subgroup of 10 “unlikely” patients with a normal neurological exam (●)

Reference values for venous lactate levels and CSF lactate levels were based on historical age-related values from our own laboratory. Normal venous lactate: 0.5 – 2.2 mmol/l; CSF lactate: 0.9 – 1.9 mmol/l. The reference value for ATP production rate from pyruvate in muscle biopsy was 42.1 – 81.2 nmol/h.mUCS. The normal MRS reference ranges for lactate (0 – 12 a.u.) and for the lactate/creatine (L/Cr) ratio (0 – 0.22) were obtained from our own MRS data. In the unlikely group, the highest value was 12 (Fig. 2a). Therefore, we defined elevated lactate as > 12. For L/Cr ratio, after correction for three “outliers” reflecting the different metabolite proportions up to 0.8 years after birth^23^, the highest value in the unlikely group was 0.22, therefore, we defined elevated L/Cr ratio as >0.22 (Fig. 2b). Blood lactate values (n = 88) and CSF lactate values (n = 61) were available from most patients. The missing values for blood lactate and CSF lactate reflect a lack of clinical indication. Venous blood was collected by venipuncture and CSF was obtained by lumbar puncture under sterile conditions. For patients from whom more than one blood or CSF sample was taken, the highest lactate level was taken.

### Statistical analyses

Descriptive statistical methods were used to assess frequency distributions and means. Because there was no normal distribution of the data, the Mann-Whitney U test was used. Differences were regarded as statistically significant when P < 0.05. All statistical tests were performed using SPSS for Windows version 20 (SPSS Omc, Chicago, Illinois USA). Although brain lactate levels are known to be higher in young infants, group analyses were not split into age categories to prevent arbitrary choices and small-group statistics.

## Results

Of the 88 patients included, 34 were female (38.6 %) and 54 were male (61.4 %). Mean age was 3.9 years (see Table 2). MRS lactate values > 12 a.u. differentiated significantly between the definite group (4/17, 24 %) and the unlikely group (0/44, 0 %, p = 0.015). No other significant differences were found between groups (Table 3). MRS L/Cr ratio values (>0.22) also differentiated significantly between the definite group (9/17, 53 %) and the unlikely group (3/44, 7 %, p = 0.001). There was also a significant difference between the definite group and the MRS reference subgroup defined as unlikely with a normal neurological exam (1/10, 10 %, p = 0.04). MRS L/Cr ratio values (>0.22) differentiated significantly between the probable group (5/10, 50 %) and the MRS reference subgroup (1/10, 10 %, p = 0.03). No other significant differences were found between groups. Note that at age under 0.8 years there were respectively 3/3, 1/2, 2/2 and 3/8 patients with L/Cr > 0.22 in the definite, probable, possible, and unlikely patient groups, and just two definites and one probable with lactate > 0.12 (Fig. 2).

**Table 3:** Incidence of elevated MRS lactate, MRS L/Cr ratio, CSF lactate values and venous lactate values

MRS values	Definite	Probable	Possible	Unlikely
	(n = 17)	(n = 10)	(n = 17)	(n = 44)
MRS lactate	4 (24 %)^a^	3 (30 %)	1 (6 %)	0 (0 %)
>12				
MRS L/Cr ratio	9 (53 %)^b^	5 (50 %)^c^	7 (41 %)	3 (7 %)
>0.22				
CSF values	Definite	Probable	Possible	Unlikely
	(n = 12)	(n = 9)	(n =15)	(n = 25)
CSF lactate	4 (33 %)	2 (22 %)	1(7 %)	2 (8 %)
>1,9 mmol/l				
Serum values	Definite	Probable	Possible	Unlikely
	(n = 17)	(n = 10)	(n = 17)	(n = 44)
Venous lactate	9 (53 %)^d^	3 (30 %)	6 (35 %)^e^	10 (23 %)
>2.2 mmol/l				

MRS, magnetic resonance spectroscopy; L/Cr, lactate/creatine; CSF, cerebral spinal fluid. Significant differences between groups: ^a^definite vs.unlikely, p = 0.015, ^b^definite vs. unlikely, p = 0.001,, ^c^probable vs. unlikely, p = 0.03, ^d^definite vs. unlikely, p < 0.001, ^fe^possible vs. unlikely, p = 0.04

Remarkably, no significant differences between groups were found for the incidence of CSF lactate elevation (>1.9 mmol/l), including the definite group (see Table 3). Detailed patient characteristics of the definite mitochondrial disease group are listed in Appendix 1, and the characteristics of the unlikely mitochondrial disease group, including the MRS reference subgroup (the first 10 patients) are listed in Appendix 2. Venous lactate values were significantly higher in the definite group (p < 0.001) and the possible group (p = 0.04) in comparison with the unlikely group (Table 3).

## Discussion

The concentration of Cr in brain tissue is relatively constant and little affected by age or by the presence of pathology [18]. Conforming to common practice in clinical MRS, we therefore used it as an internal reference for calculating metabolite ratios (lactate to creatine, L/Cr), in order to get values that were potentially the most comparable between different measurements and subsequent software versions. In our own database of MRS results for the total of 88 children this approach was corroborated by respective coefficients of variance of 23, *20*, 53, 29, and 39 for Cho, Cr. Ins, NAA, and lactate (for resp. mean peak areas of 55, 47, 3, 78, and 9 a.u.).

In our study, elevated MRS lactate and MRS L/Cr ratio values were found to be more closely associated with mitochondrial disease in children than elevated CSF lactate. Our quantitative evidence that MRS brain tissue lactate can be an important clue to mitochondrial disease is in line with previous qualitative MRS studies. Lin et al. [24] evaluated MRS data of 29 generally young patients with suspected mitochondrial disease. Patients were divided over three groups based on a classification of clinical and laboratory features and 4/8 patients with definitive mitochondrial disease (50 %) had detectable brain lactate levels on MRS [24]. Among 16 patients that had possible but not proven mitochondrial disease, three patients (19 %) had increased lactate levels shown by MRS in the Lin study [24]. In another study, Dinopoulos et al. evaluated 37 children with suspected mitochondrial disease by MRS [25]. Their patients were divided into three groups according to the modified adult criteria for mitochondrial disease by Bernier et al. [26]. Of 16 patients in the definite mitochondrial disease group, 13 patients (81 %) had detectable lactate [25]. In our definite group (n = 17) four patients (24 %) had elevated MRS lactate values and nine patients (53 %) had elevated MRS L/Cr ratio values. In our probable group (n = 10) three patients (30 %) had elevated MRS lactate values and five patients (50 %) had elevated MRS L/Cr ratio values. So, our MRS lactate values were less often elevated in the definite group (24 %) compared to the above-mentioned 50 % in the study of Lin et al. [24] and 81 % of Dinopoulos et al [25]. This difference may be coincidental, reflecting the small group sizes, or may be explained by the differences in group selection and MRS acquisition and analysis methods. Lin et al. [24] estimated “the presence or absence” of lactate in 8-cm^3^ volumes of interest, and Dinopoulos et al. [25] used a similar methodology. In contrast, in our study all spectra yielded lactate peak areas, owing to our practice of summing the spectra from the entire supraventricular region of interest measured (54-73.5 cm^3^). As a consequence, their data are more sensitive than the quantitative method we used. However, in the literature it is often seen that with the application of automated quantitative methods the results of previous, more subjective evaluations cannot be reproduced fully. An example is the overestimation of white matter lesion burden in diabetes, as performed by subjective raters rather than automated lesion segmentation [27]. Furthermore, our frequency of elevated L/Cr ratio values in the definite group is similar to the incidence of MRS detectable lactate according to Lin et al. It thus appears from our data that in quantitative analysis, the MRS L/Cr ratio value is a better marker for a mitochondrial disorder than MRS lactate alone. In addition, Chi et al. [28] assessed lactate peaks on MRS in 14 children with mitochondrial disease. Among these patients, seven patients were diagnosed with Leigh syndrome, four with MELAS, one with Pearson syndrome, one with chronic progressive external ophthalmoplegia (PEO), and one with deafness dystonia syndrome. Twelve of the 14 patients (86 %) exhibited lactate peaks on the initial single-voxel proton MRS, and all of them showed abnormal MRI findings. They concluded that lactate acquisition on MRS supports a diagnosis of a mitochondrial disease, especially in children with abnormal signal changes on the brain MRI or a normal blood lactate level. In our study only two children were diagnosed with clinically recognizable phenotypes of mitochondrial disease (see Appendix 1). These two children (one with Leigh syndrome and one with Alstrom syndrome) both had elevated MRS lactate and elevated MRS L/Cr ratios. Although the number is very small, it seems that MRS lactate elevation is more frequently seen in patients with recognizable phenotypes of mitochondrial disease. Our study adds that if the phenotype of a mitochondrial disease is less recognizable, MRS brain tissue lactate and L/Cr ratios can be of diagnostic value too. Noted here is that according to a recent investigation of 21 patients suspected of mitochondrial disorder, MRS lactate can be observed in large single voxels centred on the lateral ventricles, in levels that correlate with lactate CSF concentrations [29]. Further, it can be relevant to localize the MRS in the cerebellum, especially if cerebellar ataxia is the clinical symptom [30]. In summary, previous studies [24, 25, 28, 30, 31] used the presence of lactate peaks to define elevated MRS lactate values. We calculated exact brain tissue MRS lactate values and MRS L/Cr ratios. This makes it difficult to compare previous results with our study. Still, the previous studies showed similar findings to ours, that MRS lactate value can be a diagnostic marker for the diagnosis of mitochondrial disease in children. Our study adds that in quantitative MRS evaluation, L/Cr ratio values are preferable to lactate values expressed in a.u., even when standardized to the unsuppressed water signal.

In our study no significant differences were found between the mitochondrial disease groups and the unlikely group for CSF lactate values. This contrasts with the results of Magner et al [22]. They found that 83 % of the children with mitochondrial disorders had increased CSF lactate levels. This lack of support for our data by previous findings may be explained by selection bias or the incompleteness of our data. Because of our retrospective study design, not all patients that underwent MRS studies also underwent lumbar punctions. With respect to our results we think that elevated MRS-measured brain tissue lactate, and even more so MRS L/Cr, are better predictors for mitochondrial disease than laboratory-measured CSF lactate.

Looking at the venous lactate, we found that of the 17 children in the definite mitochondrial disease group, 53 % (nine patients) had elevated venous lactate values (Table 2). In the combined possible, probable, and definite mitochondrial disease groups (n = 44), only 41 % (18 patients) had elevated venous lactate values. These findings are supported by the work of Munnich et al. [5], who examined 1000 children who had been referred for investigation of their mitochondrial disorders. They found an elevated venous lactate value in only 30 % of the 234 patients. Given the large size of that study, their result is likely to be representative of the prevalence of lactic acidosis in children with mitochondrial diseases, and indicates that even up to 70 % of children with mitochondrial diseases can have a normal venous lactate value [5]. Also, three children in our study showed elevated MRS L/Cr values without elevated venous lactate. This suggests that MRS tissue lactate may be an even better diagnostic marker for mitochondrial disease in children than venous lactate, and needs further study. Given the many confounders that can give rise to elevated venous lactate, venipunctures often need to be repeated to gain certainty as to whether it supports the presence of a mitochondrial disease.

Limitations in this study include that not all patients underwent a lumbar puncture to determine CSF lactate values. Another limitation is that patients and not controls were used as an unlikely group, but this was for obvious ethical reasons. A third limitation is that the study design is retrospective. However, because of the low incidence of mitochondrial disease it would take years to include enough patients in a prospective study design. A fourth limitation is that, because of the retrospective design, data for patient-related factors were obtained from patient files. This could lead to incomplete data. Other limitations relate to age variation and progression of disease when MRS scans were obtained. In order to produce a reliable result from data obtained with different MRI systems and software, we calculated the L/Cr ratio, also to reduce the influence of age-related metabolite level changes. In view of uncertainties about possible changes in the T1- and T2-relaxation times of the various metabolites with aging and disease, in this paper we did not attempt to convert the lactate levels and ratios into metabolite concentrations.

Our study also has strengths. As far as we are aware no other study of this size has defined a reference range for MRS brain tissue lactate and MRS L/Cr ratio. Another strength is our presentation of exact MRS lactate values and MRS L/Cr ratio values obtained by an automated method employing curve fitting.

We do hope that our study may be of help to colleagues in their diagnostic decisions as to whether or not more invasive, time-consuming, and/or expensive diagnostic procedures such as muscle biopsy and genetic analysis are necessary. Hopefully, these invasive diagnostic procedures can be replaced by next-generation MRI sequences in the near future, through research protocols if not routine diagnostics.

## Conclusion

What was previously suggested in qualitative MRS studies based on the subjective assessment of the presence or absence of lactate signals, has now been confirmed in automated MRS quantification of brain tissue lactate: lactate and L/Cr can serve as diagnostic markers for identifying mitochondrial disease in children, or at least provide supplementary information that enhances multimodality-based diagnosis. Elevated CSF lactate appears to be a less sensitive indicator of mitochondrial disease in children.

## Appendix 1

Table 4

**Table 4:** Definite mitochondrial disease group characteristics

No.	Age^a^	Gender	Clinical & biochemical	Molecular diagnosis	Age of death	Venous lactate	CSF lactate	MRI abnormality	ATP speed	MRS lactate	MRS L/Cr^b^
	(yr)		diagnosis		(yr)	(mmol/l)	(mmol/l)				(%)
					(n = 7)		(n = 12)		(n = 8)		
1.	0.3	Male	Leigh syndrome complex I deficiency	-	0.4	8.5	ND	2	ND	28	90
2.	0.1	Male	MD complex II & III deficiency	-	-	1.6	1.6	3	11.6	8	24
3.	1.7	Female	Alström syndrome complex I & II deficiency	ALMS 1 gene mutation	-	2.0	ND	0	ND	14	27
4.	1.2	Male	MD	POLG 1 gene mutation c.1550G > T	3.0	1.5	1.6	0	16.5	10	21
5.	1.2	Female	MD complex II & III deficiency	-	-	1.8	1.4	0	20.8	9	28
6.	5.2	Male	MD	POLG 1 gene mutation	-	3.6	1.8	0	29.3	9	18
7.	1.3	Male	MD complex I & II deficiency	-	-	3.5	1.2	4	-	8	18
8.	0.2	Male	MD complex I & deficiency	-	0.3	10.5	ND	0	9.7	18	64
9.	1.4	Female	MD complex II deficiency	-	-	1.7	1.4	1	ND	10	19
10.	4.4	Female	Alpers disease	POLG 1 gene mutation	8.2	1.9	3.0	5	ND	11	22
11	0.8	Male	MD complex I, II, III & IV deficiency	-	-	3.0	1.6	0	14.1	5	11
12	10.8	Male	MD complex II & III deficiency	-	-	2.4	2.0	0	ND	9	17
13	2.1	Female	MD complex I deficiency	POLG 1 gene mutation	2.9	10	8.8	4	20.4	10	24
14	12.6	Male	MD complex IV deficiency	-	14.2	1.3	1.5	0	ND	9	20
15	3.3	Male	MD complex I deficiency	-	5.7	2.5	2.0	0	14.1	9	23
16	2.1	Female	MD complex I deficiency	-	-	5.2	ND	5	ND	17	29
17	6.6	Female	MD complex I, II, III & PDHC deficiency	-	-	1.6	ND	4	ND	7	16

*a* age at first MRS scan; *b* MRS lactate/creatine ratio; *MD* mitochondrial disease; *ND* not determined; MRI abnormality score: 0, ‘Normal’ (no abnormalities); 1, ‘Basal ganglia abnormalities’; 2, ‘Mesencephalon and/or brainstem abnormalities’; 3, ‘Atrophy’; 4, ‘Thalamus abnormalities’; and 5, ‘a combination of the abnormalities mentioned’ (1-4), PDHC = pyruvate dehydrogenase complex abnormalities

## Appendix 2

Table 5

**Table 5:** Unlikely group characteristics

No.	Age^a^	Gender	Clinical & biochemical	Molecular diagnosis	Age of death	Venous lactate	CSF lactate	MRI abnormality	ATP speed	MRS lactate	MRS L/Cr^b^
	(yr)		diagnosis		(yr)	(mmol/l)	(mmol/l)				(%)
					(n = 2)		(n = 25)			(n = 8)	
1.	2.2	Female	Developmental delay of unknown origin	-	-	2.5	ND	1	ND	6	15
2.	0.8	Male	Failure to thrive, rickets	-	-	1.2	ND	0	ND	6	14
3.	8.2	Male	Developmental delay of unknown origin	-	-	0.9	ND	0	ND	7	15
4.	1.7	Female	Developmental delay of unknown origin	-	-	3.6	ND	0	ND	8	21
5.	9.3	Female	Raynaud’s phenomenon of unknown origin	-	-	0.6	1.9	0	60.8	7	20
6.	0.2	Male	Exercise intolerance of unknown origin	-	-	3.5	1.5	0	ND	12	26
7.	12.2	Male	Loss of skills	-	-	0.9	ND	0	ND	10	18
8.	3.2	Male	Developmental delay of unknown origin	-	-	0.8	ND	0	ND	8	19
9.	6.2	Male	Developmental delay of unknown origin	-	-	0.8	1.5	0	ND	10	19
10.	12.9	Male	Mild retardation, autism	-	-	2.0	1.5	0	ND	10	19
11.	2.0	Male	Developmental delay of unknown origin	-	-	1.0	ND	0	ND	7	17
12.	10.0	Male	Severe retardation and autism	-	-	1.4	1.8	0	ND	9	13
13.	2.9	Male	Bilateral movement disorder of unknown origin	-	-	3.3	ND	0	ND	11	20
14	0.5	Female	Cryptogenic epilepsy, perceptive hearing loss	-	-	1.2	1.7	0	ND	11	19
15	5.1	Male	Epilepsy, microcephaly	15q13.2q13.3 duplication	-	0.8	1.4	0	ND	8	11
16	4.7	Female	Epilepsia partialis continua	-	-	1.5	1.5	0	ND	0	0
17.	0.1	Female	Hypochondroplasia	FGFR3-gen mutation	-	1.7	1.2	0	ND	11	17
				c.1620C > A, p.Asn540Lys							
18.	1.6	Male	Developmental delay of unknown origin, epilepsy	-	-	1.4	1.5	0	ND	9	17
19	1.8	Female	Psychomotor retardation, epilepsy	2q16.3 deletion	-	0.7	ND	0	ND	9	18
20	3.4	Male	Cockayne syndrome	homozygous mutation ercc8	-	0.7	2.2	0	55.2	9	23
21.	0.8	Male	Suspected of Freeman-Sheldon syndrome	-	3.6	1.2	2.0	1	ND	10	13
22	2.8	Male	Epilepsy	-	-	1.5	1.5	0	ND	7	15
23.	1.1	Male	Psychomotor retardation ARX mutation	epilepsy	-	1.2	1.4	0	ND	8	17
24.	1.8	Male	Failure to thrive	-	-	2.5	ND	0	ND	8	20
25.	2.2	Male	Motor development delay, perceptive hearing loss of unknown origin	-	-	1.7	1.6	0	ND	5	17
26	4.0	Male	Developmental delay, PDD-NOS	del(15)(q11.2), del(10)(q26.3)	-	1.3	ND	0	ND	8	21
27	8.0	Female	Falling of unknown origin	-	-	2.4	ND	0	ND	8	20
28	11.7	Male	Motor retardation	-	-	0.5	ND	0	ND	7	14
29	5.4	Female	Low IQ of unknown origin	-	-	1.5	1.3	0	ND	7	11
30	6.3	Male	Epilepsy, developmental delay	-	-	1.3	1.4	0	ND	10	21
31.	10.0	Male	Retardation, ADHD	-	-	0.5	ND	0	ND	8	16
32.	15.2	Male	Developmental delay of unknown origin	-	-	2.8	ND	0	ND	12	16
33	4.6	Female	Psychomotor retardation, hairy elbow syndrome	-	-	1.1	ND	0	ND	6	17
34	0.4	Female	Tonus regulation disorder	-	-	1.1	1.6	0	ND	10	20
35.	2.7	Female	Febrile seizure	-	-	1.3	1.6	0	ND	8	19
36	0.2	Male	PROM with lung hypoplasia, failure to thrive, Peripheral pulmonary	-	-	1.0	ND	0	ND	7	23
37.	5.7	Male	Myotonia, slow motor development, exercise intolerance	-	-	2.7	1.0	0	51	10	21
38	2.5	Male	Seizures of unknown origin	-	-	3.5	1.4	0	ND	8	16
39.	4.5	Male	Retardation of unknown origin	-	-	0.6	ND	0	ND	7	15
40.	3.7	Male	Muscle weakness of unknown origin	-	-	1.5	1.4	0	ND	8	17
41.	1.4	Male	Atypical febrile seizures	-	-	0.6	1.6	0	ND	9	18
42.	13.8	Male	Exercise intolerance	-	-	0.8	ND	0	ND	7	17
43.	3.3	Female	Developmental delay, epilepsy of unknown origin	-	-	1.2	1.4	0	ND	8	16
44	0.1	Female	Congenital cardio-myopathy	-	0.1	2.4	1.6	0	22.4	8	18

*a* age at first MRS scan; *b* MRS lactate/creatine ratio The MRS reference subgroup = the first 10 cases; *ND* not determined; *PDD*-*NOS* pervasive developmental disorder not otherwise specified; *ADHD* Attention Deficit Hyperactivity Disorder; *PROM* premature rupture of membranes; MRI abnormality score: 0, ‘Normal’ (no abnormalities); 1, ‘Basal ganglia abnormalities’; 2, ‘Mesencephalon and/or brainstem abnormalities’; 3, ‘Atrophy’; 4, ‘Thalamus abnormalities’; and 5, ‘A combination of the abnormalities mentioned’ (1-4)
